# Association between plant-based dietary index and gallstone disease: A cross sectional study from NHANES

**DOI:** 10.1371/journal.pone.0305822

**Published:** 2024-06-25

**Authors:** Luyang Li, Chengli Liu, Tian Xia, Haoming Li, Jun Yang, Meng Pu, Shuhan Zhang, Yingbo Ma, Tao Zhang

**Affiliations:** 1 Postgraduate Training Base of Air Force Medical Center, China Medical University, Beijing, China; 2 Department of Hepatobiliary Surgery, Air Force Medical Center, Air Force Medical University, Beijing, China; 3 Graduate School of Hebei North University, Zhangjiakou, Hebei, China; Chung Shan Medical University, TAIWAN

## Abstract

**Background:**

The relationship between plant-based diets and gallstone disease has been debated. This study aimed to shed light on the association between plant-based dietary index and the risk of developing gallstone disease.

**Methods:**

Eligible participants were selected from National Health and Nutrition Examination Survey (NHANES) 2017–2020. Three plant-based diet indexes (PDI, healthy PDI, unhealthy PDI) were calculated using data from two NHANES 24-h dietary recall interviews. Restricted Cubic Spline and multivariate logistic regression were used to analyze the associations. Subgroup analysis was adopted to make the results more robust.

**Results:**

A total of 5673 eligible participants were analyzed. After adjusting for various confounding variables, uPDI was positively associated with gallstone disease (OR = 1.53, 95%CI: 1.02–2.29). No association was found between PDI/hPDI and gallstone disease (p > 0.05). The results of subgroup analysis did not show any positive association between uPDI and gallstones in specific groups.

**Conclusion:**

Our study shows that the elevated uPDI are linked to a higher risk of gallstone disease.

## Introduction

Gallstone disease (GD) is one of the most common gastrointestinal disorders [1]. The prevalence and incidence of GD varies between countries and ethnicities. About 10% to 15% of Americans suffer from GD [2]. Gallstones are classified into three types: cholesterol stones, pigment stones, and mixed stones. In the western world, cholesterol stones are the most common [3]. The increased secretion of cholesterol in bile, crystallization, and reduced gallbladder contractility are linked to the development of cholesterol gallstones. The main cause of pigment gallstones is biliary tract infection. GD is usually asymptomatic. Comorbidities such as pancreatitis, cholangitis, and cholecystitis are major sources of surgical procedures and hospital admissions. These conditions place a significant financial strain on healthcare resources. It is important to address these comorbidities to reduce the burden on the healthcare system [2,4].

5Fs (forty years of age, female, fatty, fair, and fertile) are considered traditional risk factors for gallstones [5]. Multiple foods and nutrients are also associated with the risk of GD, and this association is complex. Previous research has shown that the risk of GD is negatively correlated with the consumption of vegetables and fiber, and positively correlated with high caloric, fat, and saturated fatty acid intake from meat [6]. Some research shown that vegetarian diet is a protective factor for gallstone disease [7]. However, some studies have concluded that vegetarian diets increase the risk of gallstone disease [8]. The inconsistent conclusions of these studies may be attributed to differences in the quality of plant-based diet and lack of quantitative evaluation of plant-base diet.

Plant-based dietary indexes are frequently suggested as healthy dietary alternatives due to their higher intake of plant foods and lower consumption of animal foods [9,10]. Compared with previous researches that treated plant foods equally, the Plant-based Dietary Index (PDI) further segregates plant-based diets into hPDI and uPDI categories. Studies showed that PDI can result in a lower risk of all-cause mortality and improved health conditions for individuals with disease such as hypertension, cardiovascular disease and diabetes [11,12].

In this study, we examined the association between plant-based diet, its quality and the risk of gallstone disease.

## Materials and methods

### Study design and population

**Fig 1:**
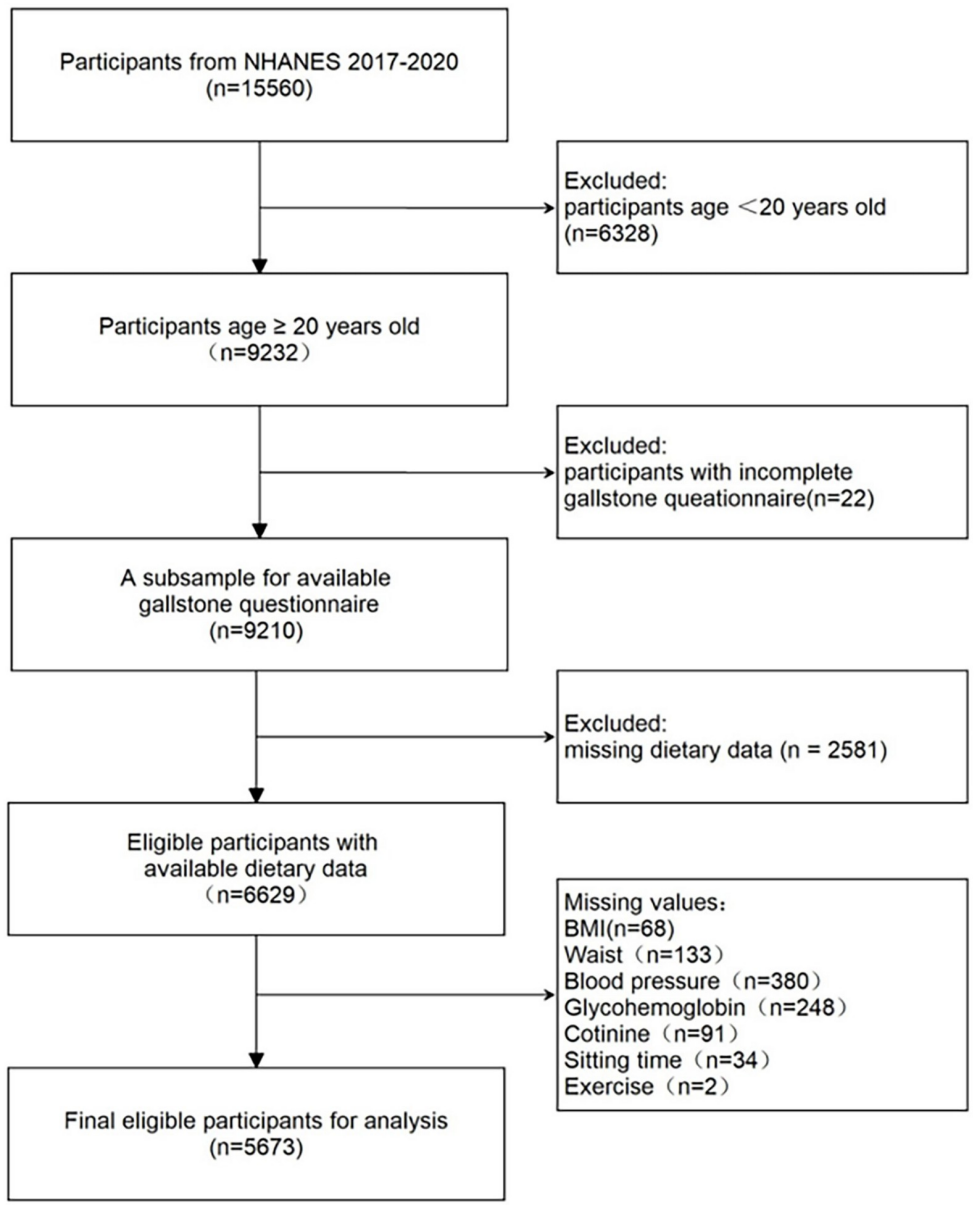
Flow chart illustrating selection of the study population in NHANES from 2017 to 2020.

A sophisticated, multistage, random sampling design is employed in the National Health and Nutrition Examination Survey (NHANES), a nationally representative study. NHANES data has been approved by the NCHS Ethics Review Board (ERB), and informed consent was obtained in writing from all participants in this national survey. In this study, we extracted data from NHANES between 2017 and 2020. In a survey conducted by trained professional interviewers, respondents were asked the question: "Has a doctor or other health professional ever diagnosed you with gallstones? " The results of the survey showed that individuals who answered "yes" were considered to have gallstones, while those who answered "no" were considered to be free of gallstone disease. Exclusion criteria were as follows: (1) participants aged < 20years old; (2) participants with incomplete gallstone data; (3) participants with incomplete two 24-h dietary recall interviews data; (4) participants with incomplete covariates. (Fig 1) shows a thorough illustration of the inclusion and exclusion procedure.

### Plant-based dietary index

PDI, hPDI, uPDI were calculated using methods similar to previous studies [11,13]. The average of two 24-hour recall NHANES interviews were used to gather dietary intake data, which were then taken from the food-pattern-equivalency database after being converted to the appropriate food equivalents. Table 1 shows the food grouping and scoring rules. Food groups were ranked into quintiles by consumption and those with highest consumption received a 5 while those with the lowest or no consumption received a 0. Likewise, reverse score is that food groups with highest consumption received a 0 and those with the lowest or no consumption received a 5. Each subject’s scores were summed to obtain a score for each index, with a theoretical range of 15 to 75. Finally, these index variables were treated as continuous and categorical (in quintiles), respectively.

**Table 1:** Calculation of three PDIs.

Food groups	PDI	hPDI	uPDI
**Healthy plant foods**			
Whole grains	+	+	-
Fruits	+	+	-
Vegetables	+	+	-
Nuts	+	+	-
Legumes	+	+	-
**Less healthy food**			
Fruit juices	+	-	+
Refined grains	+	-	+
Potatoes	+	-	+
Sugar	+	-	+
**Animal food group**			
Dairy	-	-	-
Egg	-	-	-
Fish or seafood	-	-	-
Meat	-	-	-

Abbreviations: PDI, plant-based diet index; hPDI, healthy plant-based diet index; uPDI, unhealthy plant-based diet index.

### Definition of covariates

Participants’ demographic characteristics (age, gender, ethnicity, body mass index (BMI), and waistline), lifestyle (smoking status and sitting time), and history of disease (diabetes and hypertension) were considered as covariates in the present study. Smoking status was defined by serum continine levels, which included: (i) non-smoker (<1.0 ng/mL); (ii) environmental tobacco smoke (ETS) exposure (1.0–9.9 ng/mL); (iii) current smoker (≥10 ng/mL).

### Statistical analysis

The NCHS-recommended sampling weights were employed in all analyses to take into consideration the intricate NHANES survey design. This study incorporated interview weight variables (WTINT2YRs) in all of this analyses to guarantee nationally representative estimates. To account for standard errors (SEs) associated with complex survey designs, this study utilized the primary sampling unit variable (SDMVPSU) and the stratification variable (SDMVSTRA). Continuous variables were reported as medians and interquartile ranges (25th and 75th percentiles), analysis was performed with ANOVA. Categorical variables were presented as absolute variables and frequencies, analysis was performed with the Chi-square test. Restricted cubic splines were used to flexibly model the association between PDI and the risk of gallstone disease. Survey-weighted multivariable logistic regression models were used to investigate the relationship between PDI and the risk of gallstones. A difference was considered statistically significant if it was p < 0.05, and all statistical tests were two-tailed. All analyses were performed using R software (4.1.0, R core team).

## Results

### Participant characteristics

A total of 5673 participants were included in this study. The mean age of participants was 48 years old. Among them, there were 48.76% male and 51.24% female. The differences in age, gender, race, waist, BMI, diabetes and hypertension between the non-gallstones and gallstone group were significant (p < 0.05). Participants in the gallstone group have a larger waist circumference, a higher BMI, and a higher incidence of hypertension. The majority of them were women. Table 2 presents the detailed participant characteristics. The mean scores of PDI, hPDI and uPDI were 39.8 ± 5.1, 40.8 ± 6.1, and 38.9 ± 5.4, respectively. Participants over 80 years old, Mexican American, those with BMI between 25 and 29.9 Kg/m^2^, those without diabetes, those without hypertension, and non-smoker had higher PDI. Female had higher hPDI compared with male. Participants between 20 and 40 years old, Non-Hispanic Black, those with BMI below 18.5 Kg/m^2^, those without diabetes had higher uPDI. The detailed scores were shown in Table 3.

**Table 2:** Baseline characteristics of eligible participants.

Characteristic	Overall (N = 5,673)	Non-Gallstones (N = 5,075)	Gallstones (N = 598)	p-Value
**Age (years)**	48 (33, 62)	47 (32, 61)	59 (46, 70)	<0.001
**Gender**				<0.001
Male	2,759 (48.76%)	2,585 (51.27%)	174 (27.68%)	
Female	2,914 (51.24%)	2,490 (48.73%)	424 (72.32%)	
**Race/ethnicity**				0.045
Mexican American	635 (8.00%)	565 (8.08%)	70 (7.26%)	
Non-Hispanic Black	1,528 (10.86%)	1,407 (11.27%)	121 (7.50%)	
Non-Hispanic White	2,104 (63.65%)	1,825 (62.84%)	279 (70.44%)	
Other	1,406 (17.49%)	1,278 (17.81%)	128 (14.80%)	
**Waist (cm)**	99.80 (89.40, 111.00)	99.20 (88.50, 110.00)	106.40 (95.55, 119.51)	<0.001
**BMI (Kg/m^2^)**				<0.001
< 18.5	68 (1.00%)	67 (1.11%)	1 (0.03%)	
18.5–24.9	1,316 (24.50%)	1,250 (25.86%)	66 (13.18%)	
25–29.9	1,755 (31.81%)	1,600 (32.11%)	155 (29.27%)	
≥ 30	2,534 (42.69%)	2,158 (40.92%)	376 (57.51%)	
**Diabetes**				<0.001
No	4,594 (85.82%)	4,184 (87.03%)	410 (75.65%)	
Yes	1,079 (14.18%)	891 (12.97%)	188 (24.35%)	
**Hypertension**				<0.001
No	3,054 (60.60%)	2,830 (62.92%)	224 (41.22%)	
Yes	2,619 (39.40%)	2,245 (37.08%)	374 (58.78%)	
**Smoking status**				0.400
Non-smoker	4,086 (74.25%)	3,630 (74.03%)	456 (76.06%)	
ETS	208 (3.08%)	189 (3.22%)	19 (1.91%)	
Current smoker	1,379 (22.67%)	1,256 (22.75%)	123 (22.02%)	
**Sitting time**	300 (180.00, 480.00)	300 (180.00, 480.00)	360 (240.00, 480.00)	0.300
**PDI**				0.500
Q1	1,160 (20.18%)	1,036 (20.37%)	124 (18.54%)	
Q2	1,592 (27.43%)	1,412 (27.04%)	180 (30.68%)	
Q3	1,224 (21.60%)	1,114 (21.93%)	110 (18.82%)	
Q4	1,697 (30.80%)	1,513 (30.66%)	184 (31.96%)	
**hPDI**				>0.900
Q1	1,406 (22.37%)	1,265 (22.53%)	141 (21.02%)	
Q2	1,367 (23.73%)	1,224 (23.70%)	143 (24.02%)	
Q3	1,321 (23.62%)	1,183 (23.70%)	138 (22.99%)	
Q4	1,579 (30.27%)	1,403.00 (30.07%)	176.00 (31.97%)	
**uPDI**				0.300
Q1	1,194 (24.58%)	1,074 (25.08%)	120 (20.43%)	
Q2	1,447 (27.90%)	1,297 (28.00%)	150 (26.99%)	
Q3	1,553 (24.89%)	1,383 (24.50%)	170 (28.12%)	
Q4	1,479 (22.63%)	1,321 (22.41%)	158 (24.45%)	

Abbreviations: BMI, body mass index; ETS, environmental tobacco smoke; PDI, plant-based diet index; hPDI, healthy plant-based diet index; uPDI, unhealthy plant-based diet index.

**Table 3:** The scores of dietary patterns in different subgroups.

Variables	PDI	hPDI	uPDI
**Total participants**	39.8 ± 5.1	40.8 ± 6.1	38.9 ± 5.4
**Gallstones group**			
Non-gallstones	39.8 ± 5.2	40.8 ± 6.2	38.9 ± 5.5
Gallstones	39.7 ± 5.0	41.1 ± 5.9	39.1 ± 5.3
**Age (years)**			
20–40	**39.5 ± 5.2**	**39.8 ± 6.1**	**39.7 ± 5.6**
41–60	**40.0 ± 5.1**	**40.8 ± 6.3**	**38.8 ± 5.5**
61–80	**39.8 ± 5.2**	**41.7 ± 5.9**	**38.2 ± 5.2**
>80	**40.3 ± 4.8**	**41.7 ± 5.6**	**38.7 ± 4.9**
**Gender**			
Male	39.8 ± 5.2	**39.7 ± 6.1**	38.9 ± 5.4
Female	39.8 ± 5.1	**41.9 ± 6.0**	38.9 ± 5.4
**Race/ethnicity**			
Mexican American	**40.7 ± 5.2**	**41.1 ± 5.8**	**38.1 ± 5.3**
Non-Hispanic Black	**39.2 ± 4.9**	**39.2 ± 6.1**	**40.5 ± 5.2**
Non-Hispanic White	**39.8 ± 5.1**	**41.1 ± 5.9**	**38.5 ± 5.4**
Other	**40.0 ± 5.4**	**41.9 ± 6.3**	**38.0 ± 5.5**
**BMI (Kg/m2)**			
< 18.5	**39.7 ± 5.2**	**39.4 ± 6.5**	**40.5 ± 6.0**
18.5–24.9	**40.1 ± 5.3**	**41.1 ± 6.4**	**38.7 ± 5.7**
25–29.9	**40.3 ± 5.3**	**41.5 ± 6.2**	**38.5 ± 5.4**
≥ 30	**39.3 ± 4.9**	**40.2 ± 5.9**	**39.2 ± 5.3**
**Diabetes**			
No	**39.9 ± 5.2**	**40.7 ± 6.2**	**39.0 ± 5.5**
Yes	**39.4 ± 5.1**	**41.4 ± 5.9**	**38.4 ± 5.2**
**Hypertension**			
No	**40.0 ± 5.3**	40.8 ± 6.2	38.8 ± 5.6
Yes	**39.6 ± 5.0**	40.8 ± 6.1	39.0 ± 5.2
**Smoking status**			
Non-smoker	**40.2 ± 5.2**	**41.5 ± 6.1**	**38.2 ± 5.4**
ETS	**39.0 ± 5.3**	**38.9 ± 6.0**	**40.7 ± 5.2**
Current smoker	**38.7 ± 4.8**	**39.1 ± 5.9**	**40.6 ± 5.1**

Note: A statistically significant difference was defined as p < 0.05 and data with p values below 0.05 are presented in bold type. Abbreviations: BMI, body mass index; ETS, environmental tobacco smoke; PDI, plant-based diet index; hPDI, healthy plant-based diet index; uPDI, unhealthy plant-based diet index.

### Correlation between PDI, hPDI, uPDI and gallstones

**Fig 2:**
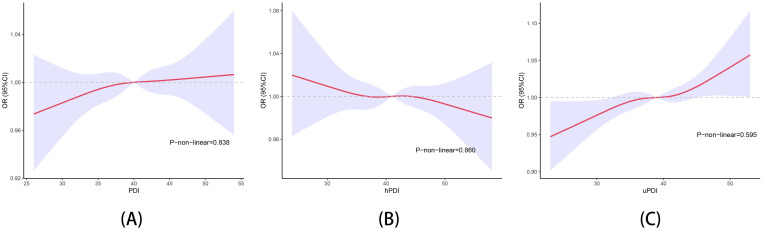
Dose-response association of three plant-based dietary index and gallstone disease. (A) the association of PDI and GD; (B) the association of hPDI and GD; (C) the association of uPDI and GD. Hazard ratios from restricted cubic splines were adjusted for age, gender, race/ethnicity, waist, BMI, diabetes, hypertension, smoking status and sitting time. Models accounted for National Health and Nutrition Examination Survey complex design and weights.

As is shown in (Fig 2), we used restricted cubic splines to visualize the relation of PDI, hPDI, uPDI with the odds of GD after adjusting all factors (p > 0.05).

Multivariate logistic regression results for the association of three PDI and GD were illustrated in Table 4. In the model 1, we adjusted age, gender and race. Then we further adjusted demographic characteristics and history of disease in the model 2. Finally, we adjusted all covariates in the model 3. Significant reverse associations were observed between odds of GD and hPDI in model 1 (OR_Q4vsQ1_ = 0.66, CI = 0.44–0.99). In all three models, there is a positive association between uPDI and GD(Model 1: OR_Q4vsQ1_ = 1.71, CI = 1.23–2.39; Model 2: OR_Q4vsQ1_ = 1.57, CI = 1.08–2.29; Model 3: OR_Q4vsQ1_ = 1.53, CI = 1.02–2.29;p for trend <0.05).

**Table 4:** Odds ratio (95%CI) for gallstones by quartiles of dietary patterns and as a continuous variable among eligible participants.

Dietary patterns	Q1	Q2	Q3	Q4	As continuous variables	P for Trend	p-Value
**PDI**							
Model 1	1.00	1.29 (0.83,2.00)	1.00 (0.58,1.74)	1.14 (0.96,1.87)	1.00 (0.96,1.04)	0.868	>0.900
Model 2	1.00	1.25 (0.80,1.94)	1.08 (0.59,1.98)	1.24 (0.75,2.06)	1.01 (0.97,1.05)	0.497	0.600
Model 3	1.00	1.28 (0.79,2.08)	1.12 (0.58,2.15)	1.32 (0.74,2.34)	1.01 (0.97,1.05)	0.385	0.500
**hPDI**							
Model 1	1.00	0.78 (0.55,1.10)	0.69 (0.41,1.15)	**0.66 (0.44,0.99)**	0.98 (0.95,1.00)	0.065	0.087
Model 2	1.00	0.83 (0.56,1.24)	0.76 (0.43,1.35)	0.86 (0.54,1.38)	0.99 (0.96,1.02)	0.485	0.600
Model 3	1.00	0.84 (0.55,1.28)	0.77 (0.41,1.42)	0.87 (0.52,1.45)	0.99 (0.96,1.03)	0.517	0.700
**uPDI**							
Model 1	1.00	1.21 (0.81,1.82)	**1.52 (1.02,2.28)**	**1.71 (1.23,2.39)**	**1.04 (1.01,1.07)**	0.004	0.005
Model 2	1.00	1.20 (0.74,1.96)	1.38 (0.91,2.11)	**1.57 (1.08,2.29)**	**1.03 (1.01,1.06)**	0.025	0.024
Model 3	1.00	1.19 (0.71,1.98)	1.35 (0.87,2.10)	**1.53 (1.02,2.29)**	**1.03 (1.00,1.06)**	0.048	0.044

Note: Values are odds ratios (95%CI) from logistic regression. Model 1: adjusted for age, gender, race/ethnicity; Model 2: Model 1 plus waist, BMI, diabetes, and hypertension; Model 3: Model2 plus smoking status, and sitting time; a statistically significant difference was defined as p < 0.05 and data with p values below 0.05 are presented in bold type. Abbreviations: OR, odds ratio; 95% CI, 95% confidence interval; PDI, plant-based diet index; hPDI, healthy plant-based diet index; uPDI, unhealthy plant-based diet index.

Then we further explore the association between uPDI and GD in different subgroups. Table 5 presents the logistic regression results and reports of a linear trend test (P for trend) in different age, gender, race, BMI, smoking status subgroup. There is no significant interaction between uPDI and other variables.

**Table 5:** Subgroup analyses of the association between quartiles of uPDI and gallstones.

	uPDI				p for Trend	p for Interaction
	Q1	Q2	Q3	Q4		
**Age (years)**						0.828
20–40	1.00	1.86 (0.35,9.80)	1.94 (0.54,6.98)	2.18 (0.60,7.87)	0.200	
41–60	1.00	1.06 (0.42,2.64)	1.21 (0.64,2.29)	1.05 (0.58,1.92)	0.700	
61–80	1.00	1.06 (0.59,1.91)	1.28(0.65,2.53)	1.70(0.77,3.74)	0.140	
>80	1.00	2.45 (0.71,8.46)	1.08(0.32,3.64)	1.27(0.31,5.20)	>0.900	
**Gender**						0.794
Male	1.00	1.01 (0.42,2.44)	1.59(0.55,4.58)	1.73(0.67,4.50)	0.140	
Female	1.00	1.28 (0.67,2.43)	1.24(0.78,1.97)	1.41(0.97,2.05)	0.120	
**Race/ethnicity**						0.132
Mexican American	1.00	0.65 (0.10,4.08)	2.05(0.39,10.9)	0.90(0.12,6.77)	0.600	
Non-Hispanic Black	1.00	1.25 (0.52,3.01)	1.85(0.76,4.48)	0.99(0.36,2.69)	0.800	
Non-Hispanic White	1.00	1.32 (0.71,2.44)	1.18 (0.69,2.00)	1.59(0.94,2.69)	0.130	
Other	1.00	0.93 (0.45,1.95)	1.62 (0.76,3.45)	2.25(0.93,5.45)	0.043	
**BMI (Kg/m2)**						0.112
18.5–24.9	1.00	2.10 (0.55,7.98)	1.41 (0.54,3.66)	0.79 (0.23,2.70)	0.500	
25–29.9	1.00	1.51 (0.67,3.38)	1.11 (0.37,3.31)	1.95 (0.88,4.35)	0.200	
≥ 30	1.00	0.77 (0.41,1.45)	1.33 (0.81,2.17)	1.40 (0.82,2.38)	0.063	
**Smoking status**						0.394
Non-smoker	1.00	1.11 (0.71,1.76)	1.35 (0.83,2.18)	1.64 (0.99,2.70)	0.048	
ETS	1.00	4.32(0.15,127.00)	4.87 (0.19,125)	22.5 (1.50,337)	0.031	
Current smoker	1.00	1.85 (0.63,5.44)	1.29 (0.51,3.22)	0.96 (0.41,2.22)	0.700	

Multivariable logistic regression models were adjusted for age, gender, race/ethnicity, waist, BMI, diabetes, hypertension, smoking status and sitting time. Stratification variables were not adjusted in the corresponding models. Abbreviations: uPDI, unhealthy plant-based diet index; BMI, body mass index; ETS, environmental tobacco smoke.

## Disscussion

Diet has always been considered a crucial factor in the formation of gallstones and the onset of gallstone related diseases. This study investigated the association of an association between three plant-based diet indexes and GD. We identified higher score of uPDI was independently associated with gallstones based on large-scale epidemiological data from NHANES. There is not such an association between PDI/hPDI and GD.

There have been several researches about the association between vegetarian diet and risk of gallstones, which are controversial between each other [7,8,14]. Research from Europe concluded that significant positive association between vegetarian diet and symptomatic gallstone disease [8]. But a cohort study from Taiwan reported that a vegetarian diet is associated with a lower incidence of symptomatic GSD in women [7]. These differences may be attributed to different population choices. The former consumes more starch. Due to the vegetarian participants were almost Buddhists, the latter consume a healthy diet (adequate amount of fiber and vegetables). Our research calculated three plant-based diet indexes which further subdivided plant-based diet into healthy and unhealthy categories while considering meat intake. So, the conclusion is more applicable to the general population. Many researches also investigated the relationship between single dietary components and gallstones [15–17]. For example, one research concluded that low fiber intake might increase the risk of cholesterol gallstones [18]. Starch consumption was positively associated with gallstones risk [7]. In our research, in the calculation of unhealthy PDI, a high score mainly represents a lower intake of meat, a lower intake of healthy food, and a higher intake of unhealthy plant-based food. Our research not only consistent with the previous research findings, but also takes into account the interactions between foods, which is more robust. Additionally, there is limited data on the correlation between overall dietary habits and the risk of GD illness. A cohort study conducted among French medical professionals found that increased adherence to MED was associated with a decreased incidence of GD disease symptoms [19]. However, non-Mediterranean countries often lack high-quality sources of MUFA due to the infrequent use of olive oil. Dena PERSIAN’s research suggests that a proinflammatory diet is linked to a lower risk of GSD [20]. In contrast to these dietary regimens, PDI is considered to be more practical.

The exact mechanisms of gallstone formation are still unclear. The mechanisms involved in this process include increased hepatic cholesterol synthesis, elevated insulin levels, and hypersecretion of cholesterol into bile, resulting in increased biliary cholesterol saturation. A large intake of refined grains leads to a negative effect on colonic motility, and increased production of secondary bile acids. These may all be related to the increased risk of gallstone disease caused by uPDI. In addition, many diets in uPDI also belong to pro-inflammatory diets. Gallstone development can be caused by inflammation-induced changes in the metabolism of proteins and lipids, which can also impact the metabolism of cholesterol and bile acids, as well as increase bile salt levels [21].

To reduce the risk of gallstone disease, participants may consider reducing their intake of unhealthy plant-based foods, as well as lowering their risk of diabetes, obesity, and cardiovascular disease [9,10,22]. Moreover, an increasing amount of research highlights the environmental benefits of transitioning to more sustainable plant-based diets. The global adoption of plant-based diets could result in significant reductions in greenhouse gas emissions, land clearance, and freshwater consumption, particularly in high-income countries where animal protein consumption is high. Switching to a mostly plant-based diet may have significant benefits for both environmental and human health [23,24].

Our study’s advantages include the use of a large, representative sample of national data, ensuring reliable results. In our study, we took restricted cubic spline to exclude the non-linear relationship between uPDI and gallstone disease. However, several limitations should be considered. First, the NHANES cross-sectional design suggests that causal links cannot be established. Secondly, the use of 24-h dietary recall may not fully reflect habitual dietary intake of the participants since it is unable to account day-to-day variation and participants may change their diet during the follow-up periods. Thirdly, although we adjusted for multiple covariates, we were unable to control for some untested confounders and residual covariates, which could lead to confounding bias. The outcome compensated for previous studies, but further large-scale prospective cohorts are still needed for validation. Further interventional studies and experimental research are warranted to identify the impact of high quality plant-based diets on asymptomatic patients who with gallstones and patients who have undergone cholecystectomy.

## Conclusion

Our study shows that the elevated uPDI are linked to a higher risk of gallstone disease. There is no significant relationship between PDI/uPDI and gallstone diseases.

## Supporting information

S1 Table Original data. (CSV)
